# Exploring the potential of mobile health interventions to address behavioural risk factors for the prevention of non-communicable diseases in Asian populations: a qualitative study

**DOI:** 10.1186/s12889-023-15598-8

**Published:** 2023-04-24

**Authors:** Jacqueline Louise Mair, Oscar Castro, Alicia Salamanca-Sanabria, Bea Franziska Frese, Florian von Wangenheim, E Shyong Tai, Tobias Kowatsch, Falk Müller-Riemenschneider

**Affiliations:** 1 Future Health Technologies, Singapore-ETH Centre, Campus for Research Excellence And Technological Enterprise (CREATE), Singapore, Singapore; 2 Saw Swee Hock School of Public Health, National University of Singapore, Singapore, Singapore; 3 Centre for Digital Health Interventions, Department of Management, Technology, and Economics, ETH Zurich, Zurich, Switzerland; 4 Yong Loo Lin School of Medicine, National University of Singapore, Singapore, Singapore; 5 Institute for Implementation Science in Health Care, University of Zurich, Zurich, Switzerland; 6 School of Medicine, University of St.Gallen, St.Gallen, Switzerland; 7 Digital Health Center, Berlin Institute of Health, Charité – Universitätsmedizin Berlin, Berlin, Germany

**Keywords:** Digital health, Mhealth, Focus groups, Health behaviour change, Conversational agents, Chatbots, Diabetes, Depression

## Abstract

**Background:**

Changing lifestyle patterns over the last decades have seen growing numbers of people in Asia affected by non-communicable diseases and common mental health disorders, including diabetes, cancer, and/or depression. Interventions targeting healthy lifestyle behaviours through mobile technologies, including new approaches such as chatbots, may be an effective, low-cost approach to prevent these conditions. To ensure uptake and engagement with mobile health interventions, however, it is essential to understand the end-users’ perspectives on using such interventions. The aim of this study was to explore perceptions, barriers, and facilitators to the use of mobile health interventions for lifestyle behaviour change in Singapore.

**Methods:**

Six virtual focus group discussions were conducted with a total of 34 participants (mean ± SD; aged 45 ± 3.6 years; 64.7% females). Focus group recordings were transcribed verbatim and analysed using an inductive thematic analysis approach, followed by deductive mapping according to perceptions, barriers, facilitators, mixed factors, or strategies.

**Results:**

Five themes were identified: (i) ***holistic wellbeing is central to healthy living*** (i.e., the importance of both physical and mental health); (ii) ***encouraging uptake of a mobile health intervention*** is influenced by factors such as incentives and government backing; (iii) ***trying out a mobile health intervention is one thing, sticking to it long term is another*** and there are key factors, such as personalisation and ease of use that influence sustained engagement with mobile health interventions; (iv) ***perceptions of chatbots as a tool to support healthy lifestyle behaviour*** are influenced by previous negative experiences with chatbots, which might hamper uptake; and (v) **sharing health-related data is OK, but with conditions** such as clarity on who will have access to the data, how it will be stored, and for what purpose it will be used.

**Conclusions:**

Findings highlight several factors that are relevant for the development and implementation of mobile health interventions in Singapore and other Asian countries. Recommendations include: (i) targeting holistic wellbeing, (ii) tailoring content to address environment-specific barriers, (iii) partnering with government and/or local (non-profit) institutions in the development and/or promotion of mobile health interventions, (iv) managing expectations regarding the use of incentives, and (iv) identifying potential alternatives or complementary approaches to the use of chatbots, particularly for mental health.

**Supplementary Information:**

The online version contains supplementary material available at 10.1186/s12889-023-15598-8.

## Introduction

Non-communicable diseases (NCDs) are non-infectious, chronic conditions that often require long-term treatment and care, impacting on patients’ functional capacities and quality of life [1]. NCDs, such as cardiovascular disease, diabetes, or cancer, are the leading causes of death and disability globally, with more than two-thirds of all annual deaths attributed to these conditions [2]. Common mental disorders (CMDs), such as depression or anxiety, also cause significant health burdens and are highly interconnected and co-morbid with NCDs [3, 4]. Risk factors for NCDs and CMDs are diverse, including a complex combination of genetic, socioeconomic, and behavioural factors [5, 6]. Lifestyle behaviours, including tobacco and alcohol use, unhealthy diets, physical inactivity, poor sleep hygiene, or ineffective emotional regulation strategies, are key modifiable risk factors for the prevention and management of NCDs [7] and CMDs [8].

The Southeast Asia region has experienced a rapid increase in NCD-related deaths [9]. For example, Singapore has undergone rapid urbanization and economic development in recent decades [10], resulting in widespread adoption of modern lifestyles characterized by low levels of physical activity, diets high in sugars and saturated fats, and demands on personal resources associated with high levels of stress. This has contributed to the high burden of NCDs [11] and CMDs [12] in the country. Several health promotion initiatives have been introduced to support a whole-of-nation effort in reducing the prevalence of chronic conditions and their burden on health services in Singapore [13, 14]. Modifying lifestyle behaviours at a nationwide scale is, however, a difficult endeavour.

Mobile health interventions may be an effective, low-cost, and scalable solution to support individuals in adopting and maintaining a healthy lifestyle [15–18]. Smartphone-based chatbots, in particular, are a novel and rapidly growing approach to digital health in which users interacts with computer systems (‘digital coaches’) that imitate natural conversation through text or voice, with the potential to improve user experience and engagement [19–22]. Such mobile interventions could be particularly effective in Singapore given the country’s high rate of smartphone ownership [23] and initiatives to promote digital adoption [24, 25].

Despite their potential however, the effectiveness of many mobile health interventions has been hampered by low engagement and high user drop-out [26–28]. To develop engaging and effective interventions, it is important to understand the end-users’ perspectives regarding the target behaviour(s), and their perceived barriers and facilitators to mobile health interventions [29, 30]. This is highlighted in several intervention development frameworks whereby meaningful engagement with key stakeholders is conceived as an integral part of the intervention development process [31, 32]. While previous research has investigated the factors influencing the uptake and effectiveness of digital health interventions, the majority of the evidence comes from Western countries [22]. Given the effects of health interventions are often highly dependent on context [31], such findings might not apply to Asian populations. Moreover, previous studies in the literature tend to focus on clinical population subgroups [33] (e.g., cancer survivors [34]), who may have different views and perceptions compared to the general population.

Considering the aforementioned gaps in the literature, the present study aimed to: (i) explore perceptions of healthy lifestyle behaviours and mobile health interventions; and (ii) identify barriers and facilitators to using mobile interventions for health behaviour change, in Singapore.

## Methods

Focus groups were conducted during July and August 2021 as part of a wider research project aimed at developing and evaluating a new smartphone-delivered lifestyle intervention for the prevention of NCDs and CMDs in Singapore [35]. Ethical approval was obtained from the Institutional Review Boards of the National University of Singapore (NUS-IRB-2021-232) and ETH Zurich (EK-2021-N-30). The Consolidated Criteria for Reporting Qualitative Research (COREQ; [36]) was used to guide reporting (supplementary file 1).

### Sampling and recruitment

In line with recommendations [31, 32], this focus group study recruited potential end-users of a new smartphone-delivered lifestyle intervention to inform the first phases of the development process [35]. Our intended target end-users are middle-aged adults who have a higher risk of developing NCDs than younger adults and may be identified as such through the national health screening programme in Singapore [37]. Participants were thus eligible to join the focus groups if they were (i) middle-aged adults (35–55 years), (ii) English speakers, (iii) Singapore citizens or permanent residents, (iv) able to provide informed consent, (v) agreed to be audio recorded, and (vi) owned a smartphone.

Participants were recruited via mailing lists from the Singapore-ETH Centre (the institution where the research was carried out) and the Facilitators Network Singapore (a local company assisting with focus group facilitation), as well as online advertisements on the Singapore-ETH Centre website and social media. Those who were interested in participating were invited to access a link or scan a QR code that directed them to a participant information sheet. It provided details of the research team, explained the study purpose and procedure, and included a recruitment survey which collected sociodemographic information, contact details, self-rated confidence using apps and the internet, and previous experience using lifestyle apps. Recruitment surveys were reviewed for eligibility and a purposive sampling procedure was used to recruit participants, whereby those with indicators of lower socioeconomic status (SES) and those with prior experience using lifestyle apps were invited to participate first. Reasons for this sampling procedure were threefold: (i) people with lower SES are at greatest risk of developing NCDs but are among the least likely to engage with health interventions [38]; (ii) there are concerns that the digital divide could exacerbate health inequalities [39] and therefore it is important to understand the views of those with lower SES on the topic; (iii) to enrich the conversations and understand lived experiences rather than hypothetical scenarios. We aimed to conduct six focus group discussions with six participants (total n = 36), based on previous research suggesting six focus groups are sufficient to identify 90% of themes in a homogenous study population using a semi-structured discussion guide [40]. There were no prior relationships between the participants and the research team. However, some participants had taken part in previous focus group research moderated by the Facilitators Network Singapore.

### Interview procedure

The questioning routes for all groups centred on perceptions, barriers, and facilitators to (i) healthy living and (ii) digital health interventions, including mobile lifestyle applications (apps) and chatbots. The content of the topic guide was developed by one researcher (JM) based upon the research team’s experience and the Theoretical Domains Framework, an integrative framework of behaviour change constructs that can be used to systematically understand behaviour and its determinants [41], as used elsewhere [42]. Questions and probes were developed to generate discussion within the TDF domains (e.g., “beliefs about consequences”, “intentions”, “knowledge”, “behavioural regulation”) and ensure the topic guide covered a wide range of factors that potentially influence users’ uptake and engagement with mobile health interventions (supplementary file 2 – topic guide). The first version of the topic guide was reviewed and piloted by the research team and the focus group moderators prior to commencing the focus group discussions. This allowed the facilitators to fully understand the goals of the research and make minor changes to the wording of the questions to ensure good understanding by the lay participants. The piloting process was not recorded or used in the final data analysis. The topic guide and questions were also reviewed and adapted iteratively following each focus group discussion (supplementary file 2 – topic guide).

Focus groups were conducted online using a videoconferencing platform (Zoom Video Communications, Inc., San Jose, CA). Discussions were facilitated by two professional local moderators from the Facilitators Network Singapore, one male (lead) and one female (supporting), who were independent from the research team and had no personal interest in the research topic. The lead moderator explained the guidelines, set ground rules, and introduced the topics for discussion. Two scribes (AA and RK) summarised participants’ comments using the chat function and took field notes during the discussions. One lead researcher (JM) attended all focus group discussions to assist the moderator if needed but made no contribution to the discussion. The questioning was semi-structured and open-ended to encourage discussion, with probes used to solicit additional information when required. Focus groups were 90 min in duration, with 15 min allocated to introductions, guidelines, ground rules, and an ice breaker, 70 min allocated to the main discussion, and 5 min to close the session. Participants received a S$30 e-voucher as reimbursement for their time. Focus group discussions were discontinued when data saturation was achieved (no new themes were identified) based on an iterative review of the field notes following each discussion and agreement among the researchers.

### Data analysis

Focus group data were analysed using a two-step approach. First, relevant themes were identified through inductive thematic analysis [43, 44], following six iterative stages: data familiarization, code generation, theme development, review of candidate themes, theme refinement, and write up. Second, sub-themes identified were categorised as perceptions, barriers, facilitators, mixed factors, or strategies by using a deductive, direct content analysis approach [45]. Epistemologically, analyses were grounded in an essentialist/realism paradigm [46]. Based on this perspective, qualitative methods aim to provide low-inference and straightforward descriptions of the phenomenon of interest, using language that is close to the collected data (i.e., within the surface or explicit meaning of the participants’ comments, rather than at the interpretative or latent level). The Braun & Clarke [43] checklist criteria for the conduct and reporting of thematic analysis was carefully followed throughout the study (supplementary file 3 – thematic analysis checklist).

In stage 1 of thematic analysis (data familiarisation), focus groups interviews were initially transcribed automatically by the Zoom platform. Two members of the research team (BF and JN) reviewed the transcripts, together with the recordings, and corrected errors. Any difficulties in transcribing certain phrases were reviewed by two other researchers (SZ and JM). Comments from participants that had been written in the meeting chat were incorporated into the transcript. Transcripts were then uploaded to Atlas.ti V.9 to facilitate the analysis. BF and JN engaged with the focus group transcripts by reading and noting down initial thoughts and observations.

During stage 2 (code generation), BF and JN generated initial codes independently for the entire sample of focus groups transcripts. In thematic analysis, codes are short labels that represent important features of the data relevant to answering the research questions [43]. For example, comments such as ‘make the app as user friendly as possible’ or ‘the user interface has to be very, very simple’ were grouped under the code ‘user friendliness’. These initial codes by BF and JN were collated, mapped for similarities, and discussed as a group in weekly meetings. During these meetings, three members of the research team (AS, JM, OC) adopted the role of ‘critical friends’ [47], that is, reviewing candidate codes / themes and offering points for reflection and alternative explanations.

In stage 3 (theme development), the final list of codes generated by BF and JN were reviewed by AS, JM, and OC, who made suggestions on whether some codes could be merged or separated and developed an initial grouping of codes independently. BF used the team inputs to implement further changes to the list of codes and grouped the codes together into a tentative set of overarching themes and sub-themes. For the purposes of this study, themes and sub-themes were understood as a collection of similar codes that provides detail about the participants’ views on healthy lifestyle and mobile health interventions.

Stage 4 (review of candidate themes) involved a critical analysis of BF’s final list of themes and sub-themes by AS, JM, and OC, to examine whether they told a convincing story of the data (one that answer the research questions) as well as revisiting the raw data under each theme and sub-theme to ensure coherence and consistency. The whole team discussed and agreed on any revisions to the final list of themes (e.g., renaming or rearranging some of the sub-themes).

In stage 5 (theme refinement), names and descriptions for each theme / sub-theme were written up by BF and JM and discussed with the rest of the team. Where relevant, sub-themes within each of the themes were then deductively mapped according to perceptions, barriers, facilitators, mixed factors, or strategies, in order to provide further structure and meaning to the data. Finally, all authors were involved in writing up the analysis and findings.

## Results

Six focus groups, each with between 4 and 7 participants, were held virtually over a two-week period during July and August 2021. Initially we recruited 36 participants but one participant did not attend the scheduled focus group and another decided to withdraw from the discussion, therefore a total of 34 participants (mean ± SD; aged 45 ± 3.6 years; 64.7% females) were included (Table 1).

**Table 1:** Characteristics of focus group participants (n = 34)

Variables	% (n), Mean ± SD
Gender (% of females)	64.7% (22)
Age	45 ± 3.6
Ethnicity	
Chinese	82.3% (28)
Indian	8.8% (3)
Arab	5.8% (2)
Malay	2.9% (1)
Residency	
Singapore Citizen	88.2% (30)
Permanent resident	11.7% (4)
Employment status	
Working full time	61.7% (21)
Working part time	14.7% (5)
Homemaker / Housewife	8.8% (3)
Self employed	5.8% (2)
Freelancer	2.9% (1)
Student	2.9% (1)
Unemployed (able to work)	2.9% (1)
Education level	
University degree and above	79.4% (27)
Polytechnic Diploma	11.7% (4)
Other diploma and professional qualification	5.8% (2)
A Level	2.9% (1)
Confidence using apps / internet	
Very confident	55.8% (19)
Confident	38.2% (13)
Average	5.8% (2)
Mildly confident	0% (0)
Not confident	0% (0)
Smartphone type	
Samsung	32.3% (11)
Apple	23.5% (8)
Xiaomi	17.6% (6)
Huawei	11.7% (4)
Oppo	11.7% (4)
Vivo	2.9% (1)
Lifestyle apps used previously	
Healthy 365	85.2% (29)
Runkeeper	20.5% (7)
MyFitnessPal	5.8% (2)
Fitbit	5.8% (2)
Map Runner	2.9% (1)
HealthHub	2.9% (1)
Strava	2.9% (1)
Samsung Health	2.9% (1)
Zero	2.9% (1)
JEFIT	2.9% (1)
BPMonitor	2.9% (1)

### Thematic analysis

Five main themes were identified through thematic analysis: (i) holistic wellbeing is central to healthy living, (ii) encouraging uptake of a mobile health intervention, (iii) trying out a mobile health intervention is one thing, sticking to it long term is another, (iv) perceptions of chatbots as a tool to support healthy lifestyle behaviour and (v) sharing health-related data is OK, but with conditions.

#### Theme 1: holistic wellbeing is central to healthy living

Holistic wellbeing was the key overarching theme when discussing the topic of healthy lifestyle. Participants felt strongly that healthy living requires a balance between both physical and mental health, as well as activities of daily life. They viewed exercise, nutrition, and sleep as core pillars, or the foundations, of a healthy lifestyle but they also identified several other aspects of life that are important. For example, having social connections and a good support network, paying attention to emotional and mental wellbeing, being surrounded by positivity, spending time on spiritual practice, or having security and certainty in life were discussed.

*Quote: “Healthy lifestyle has two aspects, one is a physical health, which is, of course, your diet and your exercise and the other one is your emotional health, which means to be spending time with your loved one, having some ‘me time’. So, to me that is healthy lifestyle, so everything has to be in balance.” (P32, Chinese Female aged 37 years)*.

*Quote: “I would think that it is more than the physical and the food and the sleeping, for example, to me, also making meaningful connections to people around me.” (P29, Chinese Male aged 41 years)*.

Participants identified three support mechanisms that they felt would help them to live a healthy lifestyle. These were trained professionals, peers or family members, and the internet. Although professionals were perceived to be the best support for mental health, there was the view that mental health stigma prevents people from seeking their support due to concerns that a diagnosis could lead to negative consequences, for example on future employment. Instead, for mental health support, people may rely more on the internet, family and friends, or a support group.

*Quote: “for the mental health challenges right, if they are not open for professional help, I’ll encourage them to join mental health support groups, you know there’s those available where diagnosis is not required, because of the stigma.” (P5, Chinese Female aged 39 years)*.

#### Barriers, facilitators, and strategies

The identified barriers, facilitators, and strategies to healthy living are summarized in Table 2. In general, participants felt people know how to lead a healthy lifestyle, but what prevents them from implementing healthy activities is a lack of motivation, discipline, or willpower. They described competing priorities, such as work commitments and taking care of family, as taking up most of their time, with a lack of personal time to engage in healthy activities. Therefore, participants felt a better work-life balance would facilitate healthy living and perhaps allow them the time to commit to a healthy lifestyle.

**Table 2:** Themes, sub-themes, and example quotes in relation to the participants’ perceived barriers, facilitators, mixed factors, or strategies

Themes	Sub-themes			
	Barriers	Facilitators	Mixed	Strategies
**1. Holistic Wellbeing is central to healthy living**	**Lack of Motivation**	**Effort**	**Environment**	**Dietary**
	*“I think the basic thing would be procrastination because often we know what needs to be done, it stops us from doing it. Sometimes we say I’ll do it next Monday, I’ll do it next week and then next week comes and then I have an excuse so to me is not so much unknowledge, but lack of willpower.” (P25, Chinese Male aged 52 years)* *“sometimes when you’re at home, when you don’t have a trainer to help you, it’s just like, yeah, discipline is a problem, very much, you just laze – sort of laze off and like do everything so slowly that you know that you are cheating yourself but, it’s always like this internal struggle that you have, you know, yeah, so that’s mainly that.“ (P6, Chinese Female aged 39 years)*	*“I’m intentionally trying all ways to make a very conscious effort in exercising daily, eating healthily (whole grains, more fruits & vegs) as much as possible and even standing up or walking around after meals till my food digests so I won’t have a tummy.” (P5, Chinese Female aged 39 years)* *“…you also have to be mentally fit right, so you must have the motivation and drive to maintain healthy lifestyle.” (P12, Chinese Male aged 54 years)*	*“…if you want to eat out, nutrition needs are pretty hard to get, healthy food at reasonable price.” (P12, Chinese Male aged 54 years)* *“…our Singapore food doesn’t help with our diet because, although the HPB [Health Promotion Board] has this diet, half of it should be protein and one quarter of the carbs and one quarter of the fiber or something like that, a lot of times you go into a foodcourt, you go into a hawker centre, you see that 80% carb you know, then 10% fiber and 10% protein, sometimes no fiber, not even bean sprout are there, you know so yeah I think there’s something that has to change, we need to” (P32, Chinese Female aged 37 years)* *“easier availability of unhealthy food in that sense, you know ordering food is so convenient, right? So that also prevents me from eating healthy.” (P24, Indian Male aged 38 years)* *“I used to work out much more because gyms were so accessible, and then after the first lockdown, things just slowed down a lot because I couldn’t go to the gyms that I wanted, and now they allow only outdoor workouts which I don’t like because it’s just so hot in Singapore.” (P6, Chinese Female aged 39 years)* *“Singaporeans are really lucky to have access to equipment and facilities around the estate at very affordable fees.” (P17, Chinese Female aged 45 years)*	*“The fact that I eat mostly at home and that helps me food-wise, making better and healthier choices when I’m at home….that helps me, be able to control what I eat and how I eat.” (P9, Indian Female aged 42 years)* *“if I had a choice, I would want to eat healthier, order less.” (P24, Indian Male aged 38 years)*
	**Lack of Time**	**Work-Life Balance**	**Social Influence**	**Activity in Daily Routine**
	*“We are often too busy, there’s too many things we need to deal with so there’s no time to actually make conscious decisions, so we just end up eating whatever we want to eat and we just end up like sometimes we don’t have enough exercise.” (P15, Arab Female aged 37 years)* *“I think in Singapore context, it is so easy to get food delivery, to go to the Kopitiam coffee shop just right below us, so it’s obvious the tight schedule of our daily work routine that we’re not able to commit that [healthier lifestyle choices].” (P36, Chinese Female aged 44 years)* *“Sometimes I’m too stressed and too busy that I’m not able to commit to the exercise that I’ve actually scheduled”. (P31, Chinese Female aged 43 years)* *“…sometimes because of time constraints, it is easier to prepare something that is already pre-prepared, for example frozen food, rather than to cook from scratch.” (P14, Chinese Female aged 35 years)*	*“Better allocate time for self instead of working all the time so that she can find time to exercise and go to bed earlier.” (P4, Chinese Female aged 42 years)* *“healthy lifestyle means more work life balance, more rest time, less stress at work and spend more time with your family.” (P33, Chinese Female aged 45 years)* *“Working only mon. to thu. - and making 3 days weekends the norm; leisure time is part of a healthy lifestyle” (P19, Indian Male aged 48 years)* *“It may be good to have a 3–4 day work week so as to spend more time with family, cultivate happiness, relax and peace.” (P21, Chinese Male aged 47 years)*	*“Social pressure. So, a lot of things have to do with who you are with. Let’s say if you’re with a group of friends who like to eat hawker food and they like to over order. Then you end up with like ten plates of all sorts of things and you’re like, oh, you know, inside your Chinese, your Asian mind kicks in that I cannot waste the food. So, they end up eating all that stuff.” (P11, Chinese Female aged 37 years)* *“It is also family, so I’ve been trying for many, many years to get my family to eat brown rice and not eat white bread right? And it’s not possible because they say Oh, you know I’m eating white rice for the whole my life.” (P12, Chinese Male aged 54 years)* *“I love walking in the evening but kids are home and I can’t go long walks…maybe after their exams” (P1, Chinese Female aged 46 years)* *“Let’s say if you’re with friends who exercise a lot, then you tend to go and exercise with them, you know but it’s a lot about getting the social network to do it with you well.” (P11, Chinese Female aged 37 years)*	*“I could wake up earlier to exercise before I start work.“ (P4, Chinese Female aged 42 years)* *“exchange my travelling time for exercise time.” (P32, Chinese Female aged 37 years)* *“in the morning, if I can squeeze some time, I might do some yoga before I start [work]. And then during lunch, normally I don’t cook so I try to walk out, go out, walk around a bit, buy my lunch, and then I come back and I continue working and then I make my own coffee. And then, at the end of the day, either someone from a family brings dinner home, if not I might try to fit in a walk so that I can just walk more and get my dinner.” (P4, Chinese Female aged 42 years)* *“go back to physical shopping, which is a way that I make myself move around more physically” (P13, Chinese Female aged 49 years)* *“not sit down for long hours after meals.“ (P5, Chinese Female aged 39 years)*
	**Ageing**			**Psychological approaches**
	*“…because all of us as we get older, we get quite a few additional pre-existing conditions, I wouldn’t say pre-existing conditions, but we get injuries and stuff like that, so you would need somebody who is actually trained to tailor a fitness experience for you, that will not aggravate your current existing injuries, you know. That’s I think one of the things why people don’t go for it, I know I would love to do HIIT, but every single time I try it, that’s it, my back goes, and you know what, never mind, forget it.” (P11, Chinese Female aged 37 years)* *“I used to run a lot, so to me, getting old stops me from having exercising you know more rigorous way.“ (P20, Chinese Male aged 54 years)*			*“set fitness goals” (P14, Chinese Female aged 35 years)* *“Meditation for good sleep” (P12, Chinese Male aged 54 years)* *“Mentally, I try not to let anger or emotions eat into me through prayer.“ (P17, Chinese Female aged 45 years)* *“Quality sleep to me is more important than duration of sleep. if there are some things on the mind before sleeping, can get them out by writing them down. With nothing bugging me in the mind, I can have a good restful sleep” (P13, Chinese Female aged 49 years)*
	**Cost**			**Time management**
	*“…they could just lower the price of brown rice and salad.” (P30, Chinese Female aged 39 years)* *“…you know once it comes to food and more interesting exercise, then I think that’s where a lot of the cost element comes into play in our society today.” (P18, Chinese Female aged 48 years)* *“I always hope that one day we can get healthier choices at a reasonable price.” (P32, Chinese Female aged 37 years)* *“nutrition needs are pretty hard to get…healthy food at reasonable prices.“ (P12, Chinese Male aged 54 years)*			*“Exercise timing I think must plan beforehand.” (P35, Chinese Male aged 42 years)* *“Develop a fixed schedule and stick to it.” (P12, Chinese Male aged 54 years)*
**2. Encouraging uptake of a mobile health intervention**	**User Type**	**Accessibility**	**Reaching people**	**Recruitment**
	*“because my habit is I don’t wear a watch, so it’s very hard for me to – I tend to forget to bring the tracker out, so I tend to not log in anything. So, tracker is not good for those who are not used to wearing watches.” (P1, Chinese Female aged 46 years)* “*I’m a bit of a tech dinosaur…I did not grow up with a phone, a smartphone, I’m just not used to things like this, and there are things like run tracker and all that, but I’m not* – *I don’t like running or cycling with a phone near me, especially running because it’s like extra weight, and I feel the one thing that prevents me from using Apps is that there’s an extra step of having to input the things.” (P6, Chinese Female aged 39 years)* “…*it’s really those who are not keen or kind of less receptive of such app will be those who are not that tech savvy.” (P36, Chinese Female aged 44 years)* *“…because it’s app-based, therefore it’s only useful if you are very comfortable using an app, or if maybe you’re already wearing tracker.” (P4 Chinese Female aged 42 years)*	*“what makes it [National Steps Challenge] a success, I think, number one is the user platform, that is accessible to everyone through the mobile phone and HPB [Health Promotion Board] even provides a free tracker if you don’t have a smartwatch that works with that, plus the logic is actually very simple.“ (P25, Chinese Male aged 52 years)*	*“some are actually quite good, like the one that is on the radio that’s like ‘quarter, quarter, half’, you know, and balance is easy and the one you just by listening to the jingle it kind of gets into your brain and then you start to notice, I actually started to look at how I ate.” (P11, Chinese Female aged 37 years)* *“I tend to only participate when there is road show.” (P1, Chinese Female aged 46 years)* *“It takes a lot of effort to go and find out about them [health promotion programmes].” (P11, Chinese Female aged 37 years)* *“I feel that the government needs this so-called ‘propaganda’, don’t be discouraged if there’s not enough converts at the moment because I want to say this is actually a lifelong process, so if it is not successful or the pickup is not high for the first two years, just keep on doing. I think some hard converts will probably pick it up after 10 years later, maybe they feel that they are not ready to do exercise, but maybe 10 years later, you do it, so I think this ‘propaganda’ should continue because it’s very helpful, some people just need this persistent calling.“ (P10, Chinese Male aged 53 years)*	*“at the supermarket, because you see that is the place where you can catch me because I go there very often.” (P1, Chinese Female aged 46 years)* “*I think people pay more attention to things that come to them in a space that they trust, that this information is useful to me, and this information is useful to my type of people so, engaging schools, if you are looking at younger crowd, engaging workplaces if you’re looking at the work crowds, getting connected with HPB [Health Promotion Board] and Ministry of Health to give the app the clout and the respectability it needs, because there are so many apps, and everything says that it’s going to change your life so, after you pay for it two weeks later, so you know it kind of [laughs] doesn’t work.” (P9, Indian Female aged 42 years)* *“you need to go through some channels to encourage the people to use it, like maybe from the company or from doctors or what they encourage the patient or employee to use it and maybe they give them some incentive or something to make them use it, at least for the first time they try it out.” (P35, Chinese Male aged 42 years)* *“It really comes to peer influence. Or maybe certain support groups to introduce such an app and how it would be able to integrate in their lifestyle, to make it see not as a chore but more of a convenience or helping them to be better every day.” (P36, Chinese Female aged 44 years)*
	**Corporate Links**	**Incentives**	**Cost**	
	*“I have noticed that it is* *based on tie ups between merchants and HPB [Health Promotion Board] so, for example, you can get points, if you buy a bubble tea, with less sugar, but you do not get points when you buy clean water from supermarket, which actually defeats the purpose.” (P14, Chinese Female aged 35 years)* *“I think one of the previous HPB [Health Promotion Board] activity long ago I saw it was actually sponsored by Coca Cola, if you figured, I did not much after that.” (P5, Chinese Female aged 39 years)*		*“…the useful ones are not free, and yeah the free ones are just not feature rich enough for you, for you to want to continue using them much.“ (P6, Chinese Female aged 39 years)* *“then they start charging, and the fee keeps increasing, the fee. Then I stop it.“ (P21, Chinese Male aged 47 years)* *“people are motivated by it [health promotion programme in Singapore] because there’s no cost involved.“ (P24, Indian Male aged 38 years)* *“seeing a professional in person is costly so if an app is providing the service at a cheaper rate that might help.“ (P1, Chinese Female aged 46 years)*	
**3. Trying out a mobile health intervention is one thing, sticking to it long-term is another**	**Technical issues**	**Tracking**	**Tailoring**	**Technical features**
	*“I also need to sync my watch to the app, and very often a lot of syncing has problems and then I sort of chuck it aside and stop syncing.“ (P3, Chinese Female aged 48 years)* *“Step tracker is supposed to be that you clock the steps that you make by walking, not by shaking your hand [laughs] yeah, so that’s one very common way to abuse the system” (P2, Chinese Female aged 46 years)* *“I find that because it’s inconsistent like Google Fit will give me a very different number from this one, so I stopped trusting any of them.“ (P18, Chinese Female aged 48 years)*	*“I have a weighing scale which is really good. It helps you to track fats and all this stuff, and break down by your, I don’t know bone density and whatever. I thought that helps.“ (P23, Chinese Female aged 46 years)* *“I think another one that got me quite thrilled was this app that actually tracks your sleeping. And it seems really important for healthy lifestyles right? So it tracks the sleep that you’re getting just snoring if you’re having abnormalities in your breathing and apparently there is even an alarm that wakes you up at your lightest sleep level so that you wake up the freshest to start your new day. I think this is really cool.“ (P30, Chinese Female aged 39 years)* *“Personally, I like the relative data, how am I compared to another person. A good example that I love to see is Singapore power right? And then, in the data they always had the national average and then the average in your neighborhood. I love that relative information, so every month or the day I can see my neighbors’ [energy usage].” (P10, Chinese Male aged 53 years)*	*“You know, oh, very important, the one that you can customize so you can actually, it is like having your own widgets inside the app that you will use, I mean not everyone’s going to have like heart problems or stuff like that” (P11, Chinese Female aged 37 years)* *“most apps, at least on the App Store, very much they look towards on a global scale in a sense, there aren’t many of the food they eat locally right? It doesn’t really accurately show like how heavy it is and then how much calories it counts to me. So, I think, in that sense, it would be better if the app can actually have a more accurate portrayal of local food in that sense…the nutrition value is not accurately captured in the existing apps.” (P24, Indian Male aged 38 years)* *“perhaps you need to lose weight and I think that health program will suit you, but I think if you have special needs, a special need tailored program, I think these general programs may not be that useful…I think a person needs tailored programs or they’ll not be that effective.” (P27, Chinese Male aged 47 years)* *“having the choice, and perhaps the bespoke-ness is something that has, that is kind of lacking. It is a certain way, and if you can mould yourself to it, then it’s good and great, and if not, then that doesn’t work for you. And that’s where sometimes when people are unable to fit it into their lifestyle, they kind of abandon it.” (P9, Indian Female aged 42 years)* *“For me, initially the initiative [National Steps Challenge] was quite good, but after a while I say, I need to clock like a number of steps within like three days or five days, and when I was disrupted by children. I tend to lose track, and you know, it defeated everything that I have put in the previous days, and I sort of felt like it was a bit wasted, so well I gave up… and then even my children, when they were participating with me as well, and they have some activities in school, and certain days they are quite busy, so we couldn’t really continue throughout that timeframe, so yeah after a while we found that it really – It doesn’t really suit our lifestyle, so we stopped.” (P3, Chinese Female aged 48 years)*	*“The app must be able solve that person’s issues and not charge a high price. No side advertisements, etc. Must be available all the time.“ (P3, Chinese Female aged 48 years)* *“Can you tell me, you know… give me one small thing to do right now to help me clock 1000 steps. Then the app suggests something, you know just to give me ideas, like becoming very interactive.“ (P13, Chinese Female aged 49 years)*
	**Burden**	**Social influence**	**Rewards and incentives**	**Incentivization**
	*“I need to input my height, my weight, my symptoms and at the end of the app, bunch of doctors was giving me diagnosis. But what I realized, one thing is, they can’t dispense medication to me, so I still have to go down to the clinic. So what good is it?“ (P17, Chinese Female aged 45 years)* *“to be honest I’m sorry but it’s just too much work for me to think like what is a food and everything else you know, in order to track how much calories I eat is also hard, so in the end that’s why I gave up.” (P18, Chinese Female aged 48 years)*	*“what will make you want to stay on the app longer is, also is prevalence, which is like, if everyone else has it, you feel that you want to have it as well, so it really works that way” (P6, Chinese Female aged 39 years)* *“So for me how it is being useful is you had that bonding with your family, your friends, your kids, your husband, you know, whoever wants to join. So technically you are…you know, bringing them along to have a healthy lifestyle, walking or even jogging so you can actually complete either five or 10 kilometer walk and then you just have a map of how long you walk.“ (P26, Malay Female aged 38 years)* *“you need to make the app popular so that word of mouth or that you get more and more people use it then.” (P35, Chinese Male aged 42 years)*	*“you get an incentive you get something like the $5 [shopping] voucher…yeah you can do it through the app, I mean I’ve been doing that before this pandemic and I thought you know it’s pretty good this incentive. So, I think if you give people incentive to do this healthy activity, you know, to get $5 $10 etc. I think people will be more motivated to go for it, and then I think that it works.“ (P28, Chinese Male aged 50 years)* *“it’s very fulfilling, it’s like when I hit a goal, I get to redeem NTUC [supermarket] vouches for that yeah, so I feel that it’s like a means to keep myself healthy and motivated” (P5 Chinese Female aged 39 years)* *“I have actually seen some people who shake their steps tracker in order to achieve that 10,000 steps a day so that they can get the vouchers that they want, in a way, it is cheating and defeating the purpose.“ (P14, Chinese Female aged 35 years)* *“After I completed the challenge for the national steps thing right, I couldn’t redeem any more incentives, so I kind of lost steam a little bit.” (P5 Chinese Female aged 39 years)* *I feel like more education on why exercise is important will be more important than just, oh you like hit 10k steps, you can get $5 voucher, like, that’s not going to be sustainable, I think, and it’s like just missing the point of it,”(P6 Chinese Female aged 39 years)*	*“if we can use it on something healthier and add on more points, so is like wow, I get motivated to even pick the healthier choices and so on and so forth.“ (P13, Chinese Female aged 49 years)* *“I honestly feel that HPB’s [Health Promotion Board] model (free tracker coupled with incentives) is pretty effective and has seen a very good adoption rate” (P24, Indian Male aged 38 years)* *“I feel that there must be some motivation, as to what motivation I don’t know, probably something that links to long term… we do something now but the benefit will come 30 years later somehow whatever, maybe your [pension] will give you 1% more. Something along that line yeah, but it is a delayed gratification because it lines up with these habits, but don’t do something immediately when someone does 10k step, I give you immediate $10 cash, no, no, no, maybe give a great voucher that years later, if your still around, 30 years later, for example.” (P10, Chinese Male aged 53 years)* *“it will be a wonder if I can marry the both, meaning to say instant gratification and delayed gratification you can give me something small right now but yeah I know I’m banking into something in the future.” (P13, Chinese Female aged 49 years)* *“So it’s like it gives you an incentive to do something useful for yourself, so maybe if you want people to take up this app it’s like you give them some NCD [no-claims discount] that can be up to 30% or something like that, on your insurance, over let’s say, if you do this over like two or three years, and you know you, you get 30% off [health] insurance.” (P11, Chinese Female aged 37 years)*
				**Personalisation**
				*“For example, today, what are the things I eat that I key in, then I hope that there can be some feedback and then what should I improve on in my next meal and then also like about the mental part how I am feeling today I will key in my moods and my feelings today, then is there any help. I mean suggestion on what can I do so, I hope that or some programs that I can attend to improve myself so use the apps have some type of feedback features would be good.” (P31, Chinese Female aged 43 years)* *“What I want to see based on this app would be more on our achievements. Each time we use it, it gets better, it recognizes our achievement. Because you have to key in your bio data. The app certain, you know how fast you walk. Yes, so you’ll be more naturally motivated each time.” (P26, Malay Female aged 38 years)* *“But I think this health app would need to kind of tailor made according to the different persona that we are actually focusing at. So if let’s say the persona group they are looking at right now is youngsters, or maybe let’s say tertiary students because the secondary school and tertiary ones are the ones that use more app-savvy tried to hook onto their phones and all. Then the app’s functions would able to address their different pain points, maybe stress at O level, you know what other sources they can tap on. Which can be a hyperlink to certain music to give me stress relief or some song playlist that I can actually run to do my exercises and help me to relieve stress at the same time.” (P36, Chinese Female aged 44 years)*
				**Integration/Multi-service**
				*“actually draw data from the various apps I think like one of your said just now, you can have many expert, yet the can I mean, I have to grant access, of course for the super app and let it draw the data from such other apps.” (P13, Chinese Female aged 49 years)* *“a centralized database that allows a lot of sharing of information, so I don’t need to keep inputting whatever I am into the system over and over again.“(P18, Chinese Female aged 48 years)* *“because there are several different health apps, if they could centralize something, so that it is one app with more things inside. That might be easier for us to keep track and take note, because right now there are several different apps for different things.“ (P15, Arab Female aged 37 years)* *“I really hope that one day we have an app that can put everything together like can track your exercises like our steps your active hours, minutes, but yet at the same time, if I say you have some emotional problem anxiety issue, there is also pages for you to link there, or even have a tele-doctor or tele counsellor.” (P32, Chinese Female aged 37 years)* *“Not just a health aspect, perhaps get also help on a broader perspective, also able to maybe work on areas like sustainability. Like…when I use my own container you know you have to reduce carbon footprint at the same time, maybe it’s like a one stone can kill many birds kind of proposal, I think that might actually help to prolong the usage of the app itself, where they actually serve more than one main key function.” (P36, Chinese Female aged 44 years)*
				**Ease of use**
				*“Make the app as user friendly as possible, download and set up the app for the person, provide proper training to use the app.” (P4, Chinese Female aged 42 years)* *“Is it user friendly for most of us? Yes, but I think a large chunk of them will also be elderly folk and you need people to train them hopefully the kids but probably we need some coaches to train them as well.“ (P30, Chinese Female aged 39 years)* *“the user interface has to be very, very simple, I mean once you download an app and you find there’s a learning curve, you’re more likely to just delete it in the next 5 s, so for it to be very simple to use yet very feature rich and very robust is, I think this is important.” (P6, Chinese Female aged 39 years)*
				**Gamification**
				*“different types of challenges different type of games to bring you to keep you on track on these activities, I thought that might be something useful.” (P33, Chinese Female aged 45 years)*
				**Social support**
				*“if you can issue challenges to a friend, like, I can challenge my friend to walk 10 K for this entire week you know things like that. I think it would be fun, you know, when a group of friends come together and an issue this kind of challenge, or maybe you can say that, together, as a group, if you achieve this, then you’ll get something.“* *“I think for the rest of the group is really, have to come to peers influence. Or maybe certain support groups to was able to introduce such app yeah and how this APP would be able to integrate in their lifestyle, to make it as to actually see not as a chore but more of a convenience or in helping them to ya to be better every day.“*

Environmental factors were viewed as both barriers and facilitators to healthy lifestyle in Singapore. For example, residents generally have good access to parks and outdoor activity spaces, such as outdoor gyms, and fitness centres are generally low cost. However, the weather conditions limit outdoor activity to the early morning or evening, when temperatures are cooler. Participants also explained that the food environment in Singapore does not promote healthy eating, as unhealthy food is cheaper and highly available, compared to healthy food.

Social influences were also perceived as creating both barriers and facilitators to healthy eating and participation in exercise and physical activity. Participants talked about how food choices are influenced by what others are eating or the food preferences of others, both when dining out and preparing food at home. They also talked about how involving family and friends in physical activity can motivate them to be more active.

Participants also described the actions they were already taking or could take, to lead a healthy lifestyle. Dietary strategies included making conscious diet choices, for example eating more balanced meals, ordering less ‘take away’ food, and cooking at home. Exercise strategies focused on integrating movement into daily activities, for example walking after meals and taking activity breaks during the workday. Different psychological strategies included setting goals, following a routine, journaling, and spiritual rituals like meditation and praying. Finally, time management strategies, including a focus on work-life balance and planning ahead for healthy eating and physical activity, were named by the participants.

#### Theme 2: encouraging uptake of a mobile health intervention

Theme 2 discusses the factors influencing the initial uptake of mobile health interventions. Participants’ comments were largely in relation to digital health promotion programmes offered by the Singaporean Government’s Health Promotion Board (HPB), although opinions on other commercially available apps were also discussed. In general, participants were open to using digital health interventions and apps. They acknowledged that the Singapore Government has made significant effort to promote health through digital programmes and that the incentives offered with these programmes (e.g., offering free wearable trackers) are effective in getting people to sign up initially. Some also commented that programmes or apps that are popular, and that many people are talking about, encourage wider uptake. Apps with essential features linked to lifestyle behaviours, for example, booking systems for fitness classes, or tracking body weight with a linked digital scale, also have higher uptake.

Quote: *“I honestly feel that HPB’s model (free tracker coupled with incentives) is pretty effective and has seen a very good adoption rate.” (P24, Indian Male aged 38 years)*

Quote: *“For me, I feel probably you need to make the app more popular first, maybe some incentive to initially just jump start, like everyone now notices the [online retailer] app. You need to make the app popular so that word of mouth or that you get more and more people use it then. (P34, Chinese Male aged 42 years)*

Quote: *“I was introduced to it [Healthy 365 app] because there’s a dance class that goes on right underneath my window, and that was like so attractive, so I just appeared then they told me, oh yeah you can dance here, and you can download this app.” (P9, Indian Female aged 42 years)*

#### Barriers, facilitators, and strategies

The identified barriers, facilitators, and strategies for the uptake of mobile health interventions are summarized in Table 2. In terms of barriers, participants found that the type of user could prevent uptake, for example, people who do not like to wear watches or activity trackers or those who are less technically savvy. They also highlighted decreased trust in health programmes with corporate links or sponsorships, as they found the backing of certain companies stood in direct opposition to the proclaimed health objectives of the programmes (for example in the case of fast-food companies). One key facilitator to the uptake of mobile health interventions was accessibility. Programmes that offer a digital platform that can be accessed by anyone, regardless of demographic or background, were seen as highly inclusive and more likely to be used. As an example, access to free apps and activity trackers, as in the case of the National Steps Challenge in Singapore, was seen as an enabler to join the programme.

The marketing strategies and outreach efforts used to encourage uptake of mobile health interventions were seen as both a barrier and a facilitator. On the one hand, roadshows and the use of radio jingles were mentioned as memorable tools that convinced people to join. On the other hand, one participant said that it was often difficult to find further information about ongoing programmes and it was too effortful to sign-up to participate. Similarly, there were mixed views regarding the cost of commercial apps. While participants felt cost was a limiting factor in the uptake and long-term use of commercial apps which ultimately led the abandonment of the app, they were also dissatisfied with the limited features available with free apps. Still, if apps were able to offer the same service as comparable offline services, such as receiving support from a health professional, participants saw cost benefits for the app.

Strategies for the uptake of mobile health interventions were centred around ways to reach people. Participants viewed places visited frequently by people, such as supermarkets, schools, or workplaces, to be the best places to reach and engage people with mobile health initiatives. In addition, participants felt certain individuals, such as doctors, friends, or community support groups, would be best placed to convince people to use mobile health interventions. Consistent and persistent messaging about a mobile health intervention over a long period of time was also mentioned as a way to encourage uptake.

#### Theme 3: trying out a mobile health intervention is one thing, sticking to it long term is another

Theme 3 explores user experience and factors influencing long-term engagement with mobile health interventions. Overall, participant’s perceived mobile health interventions as useful and effective, highlighting the benefits of certain features like providing free apps and activity trackers, being able to collect rewards, and using the technology to connect with group activities and workshops.

Quote: *““Lose to Win” is also organized by the Health Promotion Board…I took part in a few seasons, and I think it was really, really helpful. The first time when I take part in it they organize you into groups and then you meet regularly to exercise together, and also on nutrition workshops and yeah talks and workshops, which is very helpful. I got to know a few friends from that programme.”* (P2, Chinese Female aged 46 years)

Participants mentioned that the novelty and excitement of a new app encouraged them to use it more in the beginning, but after some time, the novelty effect would wear off and they would either stop using the app as frequently or give up using it altogether.

*Quote: “that’s quite exciting for the first two seasons of this ‘National Steps Challenge’, then after that, even though I sign up on the latest ‘National Steps Challenge’ but because I’m not clocking much [steps], I kind of also semi give up, yeah [laughs]. So yeah, the initial excitement has gone.”* (P2, Chinese Female aged 46 years)

*Quote: “I think the initial stage, because of the novelty people will get a tracker try to clock 10,000 steps, every day, but after a number of days, it wears off, so once the novelty wears off, we are back to our usual: people who are active remain active, people who are not active will still be not active because it’s like there’s no more incentive anymore really.”* (P32, Chinese Female aged 47 years)

#### Barriers, facilitators, and strategies

The identified barriers, facilitators, and strategies linked to long-term engagement with mobile health interventions are summarized in Table 2. Participants felt technical issues, for example, problems synching apps with trackers or measurement inaccuracies, and high levels of user burden, such as when manually tracking dietary intake, were key barriers to continued engagement with apps. Tracking health and behaviours, particularly via passive sensing, and being able to compare data with others, were seen as facilitators to engagement. Additionally, social influences were identified as facilitating engagement in two ways, first in the sense that people want to use an app that everyone else is using, and second, it can be motivating when other family members and friends are using the same app and provide support to engage in healthy activities.

Rewards and incentives via mobile health interventions were seen as both facilitators and barriers to engagement with healthy lifestyle behaviours. On the one side, participants said incentives, such as receiving vouchers for performing healthy activities, can be effective in engaging people with a mobile health intervention and help to motivate them to change their behaviours. On the other side, they acknowledged that this form of extrinsic motivation is unlikely to work long-term and can lead to users abusing the system. For example, when rewards and incentives are no longer available users become disengaged, suggesting they will only perform the desired behaviours when an incentive is involved.

There were also mixed views on tailoring and personalisation of app content and features. Although tailoring was perceived as a very desirable feature, participants commented that existing apps often take a ‘one-size-fits-all’ approach and, while that approach might work for some people, it is unlikely to work for the majority. In general, if a mobile health intervention is not tailored, personalized, or bespoke to the individual, people will eventually abandon it.

Participants identified several strategies that they felt would improve engagement with mobile health interventions. Social strategies included collaborating as a group with family and friends to achieve prizes or using peer pressure to help drive behaviour change. In relation to incentives, while they were identified as creating both barriers and facilitators to longer-term engagement, participants still felt that they could be used to further facilitate long-term engagement. For example, they suggested different models of incentivisation such as delayed rewards or only allowing users to redeem points against healthier choice products. Regarding desirable features, participants highlighted the importance of personalization, whereby an app can cater directly to a user’s specific needs and tailor support or content as needed. They also discussed a desire to be able to track and visualize their progress and achievements easily. In addition, apps should be extremely easy to use, offer multiple health and wellbeing services, and integrate different data sources in one place to reduce burden on participants.

#### Theme 4: perceptions of chatbots as a tool to support healthy lifestyle behaviour

Participants’ views on chatbots were largely based on their experiences using customer service-related support. They had a negative perception of chatbots, perceiving them as useless, and described the process of interacting with them as frustrating. Specifically, chatbot interactions are time-consuming and the chatbot itself is often unable to understand questions or provide helpful answers. These issues were perceived to have a demotivating effect on users and made them sceptical about how useful a chatbot would be in the health context.

Quote: *“I think it’s just based on past or present experiences that we had on chatbots, they’re usually not answering your question directly, I think a lifestyle, digital coach, whether health exercises or even mental health, I guess, I wouldn’t want to use it.”* (P3, Chinese Female aged 48 years)

Quote: “*Well, if it is still not a real person, then after all it is programming, so I wouldn’t say that I have much confidence in it, because, after all, for everyone the problem is unique.”* (P32, Chinese Female aged 37 years)

Participants were also concerned that chatbots, or digital interventions more generally, might be used to replace face-to-face services and could lead to job losses for certain professionals, like psychologists.

Quote: *“…might people lose their job or get replaced? If you can understand yourself, why would we need a doctor, why would we need a psychologist, physically?”* (P26, Malay Female aged 38 years)

While participants were open to the idea of a chatbot supporting them with certain health issues or behaviours, there was concern that, for mental health support, a chatbot might not only be demotivating but also problematic and that AI might never be able to replace a human being.

Quote: “*One of the bad things about digital health coaching is that it can come across as slightly impersonal and the other thing is that it may try to be motivational but it might end up having a really opposite effect”* (P11, Chinese Female aged 37 years)

*Quote: “For me, I think, I would like to know whether it is a chatbot or real person. I mean if it’s for food, okay lah, just a chatbot will do. But if it’s a mental health coach, I would prefer a human being. I mean a real-life human behind the chat. I mean, that’s why I feel that robotic or AI can never replace a human being, I’ll need the touch, yeah, so I think for mental health, I prefer a human being behind it.”* (P1, Chinese Female aged 46 years)

*Quote: You can have the Doctor Google or Alexa, you know, answering your queries anytime of the day, but if you’re talking about psychological well-being it’s pretty hard to trust just a robot, you know, answers which are like generic anyway.”* (P23, Chinese Female aged 46 years)

However, they also acknowledged that the technology is still in its infancy and apps using digital health coaches could have potential due to their availability, accessibility, and cost-effectiveness when compared to offline options.

*Quote: “So I mean of course there are like some established chatbots where people use it so much that the AI is good enough to be able to give good answers, but it always takes – yeah I mean it’s a good time to start – but it always takes a while before the data collected is good enough for it to give reasonably good answers.”* (P6, Chinese Female aged 39 years)

*Quote: “compared to like a physical coach, right, I think I would be more receptive to having a digital coach. Simply because of the availability, the accessibility and then I think working with a digital coach would also be more cost effective. In a sense, I think, probably for a digital coach, at most, the cost involved probably would be from your monthly subscription if it’s chargeable in that sense. But then, whereas you know if you have a physical coach, I think in the terms of costs here will be higher. So in that sense, I think I would be more receptive towards the app.”* (P24, Indian Male aged 38 years)

Potential strategies to improve chatbots for the purpose of digital health coaching were largely focused on the qualities of the chatbot and the type of support that the chatbot could provide. For example, the chatbot should be empathetic, interactive, encouraging, and helpful, like a virtual friend who can provide motivational support and makes personalized health suggestions based on current progress. Participants also suggested giving users different ways of interacting with the chatbot, for example through speech or text, offering free trial periods to test out the chatbot, and providing an option to link users to real-life coaches.

Quote: *“…the interactive element there, so if a digital health assistant wants to maybe be a little bit more effective, it should be your friend. It shouldn’t be something that is very much like your doctor sitting there and then looking at vitals. It should be the friend who says ‘hey, wanna come with me today and let’s go for just a short walk?’ and then after that before you know it, that app has brought you on a slightly longer walk, than the short walk that you originally wanted. And then the app says ‘hey I had lots of fun with you’ and all that kind of thing, so it has to have a very human side to it in order to be effective.”*

#### Theme 5: sharing health-related data is OK, but with conditions

Participants were largely unconcerned about health-related data sharing, especially if they could see a benefit for them or the wider community. However, this sentiment was conditional on three aspects: (i) who will have access to the data, (ii) how it will be stored, and (iii) for what purpose it will be used. Participants needed to be able to trust the entity accessing and using their data. In this regard, government agencies were viewed as more trustworthy than private companies, as the latter were perceived to benefit from the data themselves by monetizing it. Participants were generally happy to share their data if it was aggregated, anonymized, and securely stored. Participants highlighted the importance of being informed about the proposed use of their data and being explicitly asked to provide their consent for this use.

*Quote: “I know I’m sharing my personal data for the better of the community.”* (P21, Chinese Male aged 47 years).

*Quote: “I think what’s important is to generate more trust or like you know, for us to want to be more willing to share data, we’d like to know, what actually is being done with the data, like, why do you need this information and what’re you going to do with it and what do I get back in return for sharing that bit of data and how it can help me?* (P6, Chinese Female aged 39 years).

## Discussion

The aim of this qualitative study was to explore perceptions, barriers, and facilitators to mobile health interventions targeting healthy lifestyle behaviours in Singapore. We identified one broad theme relating to healthy living and holistic wellbeing, two themes relating to the uptake and continued use of mobile health intervention, and two narrower themes discussing the potential of chatbots in supporting health behaviour change and in what circumstances health-related data might be shared. These themes and relevant sub-themes are discussed below, with a particular focus on the practical implications for developing and implementing mobile health interventions in Singapore and, potentially, other Southeast Asian countries.

Participants showed a generally positive attitude towards digital health interventions in general and highlighted the importance of targeting holistic wellbeing, including body, mind, connectedness, and spirituality. Current health-related interventions, however, rarely address these elements together but rather focus on a single domain such as doing more exercise [48]. Even with health promotion programmes that target different domains, there is generally a greater emphasis on combining physical activity, sleep, and diet, while other less tangible elements related to mental health and wellbeing (e.g., emotions, social relationships, life values) are hardly integrated (e.g., [49]. While further research is needed, our findings suggest that digital health interventions targeting Asian populations in Singapore might benefit from adopting a ‘body and mind’ approach, highlighting the significance of the whole human entity and the interdependence of its parts (physical, emotional, and spiritual). This coincides with contemporary views on mental health, which emphasise the care of both mind and body [50]. Face-to-face programmes targeting holistic wellbeing exist and could serve as an inspiration for future digital health interventions (e.g., The Integrative Body–Mind–Spirit (I-BMS) psychosocial programme [51].

Most of the identified barrier and facilitators to leading a healthy lifestyle were in line with previous literature in Singapore and beyond. For example, several studies have highlighted lack of time (e.g., competing priorities) and cost (e.g., affordability of healthy food or exercise equipment) as key barriers to healthy living [52–56]. Similarly, social support (e.g., pairing with someone with similar goals) has been identified as a facilitator for health behaviour change [56, 57]. There were, however, some environment-specific barriers that are unique to Singapore and other Asian countries which should be considered by future intervention development teams. One of such is tropical weather, which features hot and humid temperatures all year-round and might discourage individuals from exercising or taking part in sports activities. Mobile health interventions including a physical activity component should consider such environmental factors and tailor their recommendations and activity suggestions accordingly (e.g., prompting individuals to exercise indoor or early / late in the day).

The food environment in Singapore was also widely discussed during the focus groups, with participants agreeing it is not conducive to healthy eating as energy-dense, nutrient-poor foods are widely available, affordable, and heavily promoted. This is in line with recent studies analysing the health of the Singapore food environment [58], and it might hamper individuals’ intentions and goals related to healthy eating. In addition, diets traditionally considered as healthy and widely recommended by public health organisations such as the Mediterranean diet might not be as accessible in Singapore as they might be in Western countries, considering availability and costs (e.g., Singapore imports 90% of its food). This should be considered by mobile health intervention developers and other public health initiatives targeting healthy eating in Asian populations, with dietary suggestions tailored to the local context and variety of ethnic backgrounds (e.g., Chinese, Indian, and Malay).

A key challenge for any mobile health intervention lies in how to motivate users to start using the app and/or wearable device and adhere to it long enough [27, 30]. Participants reflected on a wide range of variables influencing uptake and engagement with mobile health interventions. Consistent with previous research, factors such as user-friendliness, personalization, gamification and social influences were suggested as facilitators to engagement [59–62], while lack of digital literacy, poor clinical workflow integration, and technical issues were highlighted as barriers [30, 33, 60, 63]. A noteworthy difference to previous studies (mostly conducted with Western populations) is the influence that government endorsement has on a users’ decision whether to start using a given digital health intervention or not. Participants were also more willing to share health-related data with government agencies compared to private companies. This is consistent with previous research showing that Singaporeans have a higher level of confidence in the government, and are more supportive of government surveillance, compared to Western countries [64]. In addition, a substantial proportion of Singaporean adults have experience participating in government-supported, nationwide mobile health interventions such as the National Steps Challenge [14], which reached approximately 26% of the adult population in Singapore between 2017 and by 2019 [65]. Taken together, these findings suggest that future mobile health interventions in Singapore might benefit from partnering with government and/or local (non-profit) institutions, as this is expected to facilitate trust (e.g., data-sharing) and maximise uptake.

The use of rewards and incentives was generally viewed by participants as a useful strategy for both initial uptake and sustained engagement with a mobile health intervention. Financial incentives in the form of redeemable points or coins are common in the Singaporean app landscape, both for health- and non-health-related apps. However, the effectiveness of financial incentives is debated in the behaviour change field [66, 67], with some arguing that they undermine intrinsic motivation, which is thought to be a key process for sustained engagement [68]. In contrast, others defend their use as a useful strategy to encourage initial uptake, leading to more self-determined forms of motivation in the long-term [69]. Participants of digital health programmes implementing a reward system have also shown mixed views on the use of incentives [70]. Regardless of the intervention developers’ position, they should consider that some sort of external reward system might be expected in Singapore and act upon this, either by incorporating incentives or focusing on expectation management. Other engagement strategies mentioned by participants that could substitute the use of incentives are gamification, use of social support, personalisation, and app integration (‘one app for everything’, in line with the demanded holistic health approach).

Chatbots are rapidly becoming important gateways to delivering digital services in multiple areas, including health, customer service, or work assistance [71]. However, participants had mostly a negative opinion towards chatbots and their potential usefulness. This might be because their experiences were limited and mostly based on artificial intelligence (AI) chatbots used in the customer service domain, which are powered by natural language processing and use machine learning to understand the context and intent of a given question before formulating a response [22, 71]. AI chatbots are complex and highly dependent on the amount and quality of the data used to train the system. Interactions with AI chatbots often fail to meet user expectations, as questions are sometimes misinterpreted or cannot be answered, and this can be a source of frustration [72]. We argue that the use of rule-based chatbots might be a better alternative for digital health interventions until AI technology matures. Rule-based chatbots are based on decision trees instead of AI, using pre-defined questions and answers throughout the interaction (e.g., [21, 73]. With a rule-based chatbot the intervention developer has a high degree of control over the conversation flow and can ensure a smooth and effective interaction. This is especially relevant in the health context where information needs to be delivered in the most accurate and comprehensive way [74].

Lastly, it is worth mentioning that participants were particularly concerned about the use of chatbots for mental health support, compared to physical activity and/or healthy diet promotion, and felt strongly that chatbots should not replace humans in this regard. These views have also been reported elsewhere [75, 76] and may stem from the perceived lack of emotional connection with a chatbot, which is a key component of mental health interventions, as well as the reluctance to disclose potentially sensitive mental health information through a digital device. Participants mentioned different types of interaction styles that the chatbot could employ (e.g., empathetic, pragmatic, interactive), which is a relatively novel research topic in the field [77, 78]. Further research is warranted to explore chatbot interaction styles and their impact on engagement, acceptance, and working alliance in specific populations, as well as identifying potential alternatives to chatbots for delivering scalable and engaging digital health interventions.

### Strengths and limitations

Strengths of this study include the use of the COREQ reporting guidelines [35] and the 15-point checklist criteria for ‘good’ thematic analysis throughout the transcription, coding, data analysis, and writing processes [43]. Specific good practices include ensuring the data have been transcribed to an appropriate level of detail, giving equal attention to each data item in the coding process, and clarifying our epistemology stance. The fact that several co-authors acted as ‘critical friends’ throughout the thematic analysis (e.g., offering critique and asking reflective questions, suggesting data to be examined through another lens, etc.) was also a key strength as it allowed the main analysts to think more deeply about the data and consider alternative explanations and groupings of codes. We believe this led to a richer, more comprehensive account of our participants’ perceptions and experiences of healthy lifestyles and mobile health interventions.

Our study also has some limitations that need to be considered. Results are based on a predominantly Chinese sample with a majority of women. Therefore, findings may not be applicable to all middle-aged adults in Singapore. Despite our purposive sampling procedure prioritising individuals with low SES, nearly 80% of our participants had a university degree, which is a strong indicator of middle-to-high SES. There is a steep socioeconomic gradient for both the incidence of NCDs [79] and engagement with digital health interventions and other health promotion initiatives [33]. Therefore, it is important that people with low SES are actively recruited and take part in future formative studies. Additionally, the purposive sampling procedure to recruit those with prior experience using lifestyle apps, and the fact that most of the participants felt confident or very confident using apps and the internet (as outlined in Table 1), means the opinions described in this paper are drawn from people who are comfortable using such mobile interventions. Furthermore, the specific recruitment strategy used in our study involved an active role from the participant, who had to contact the research team to participate in the study. Therefore, it is possible that the participants who took part were those already interested in health research or engaged in the topic. Finally, we did not gather data on health status and therefore we do not know whether participants’ opinions were influenced by existing health conditions.

## Conclusion

This study adds to the limited body of literature exploring public perceptions of mobile health interventions in Asian populations. Findings highlighted several factors that are relevant for promoting a healthy lifestyle and for the effectiveness of existing and emerging mobile health interventions in Singapore and other southeast Asian countries. Future work in this area should consider: (i) targeting holistic wellbeing, (ii) tailoring content to address environment-specific barriers, (iii) partnering with government and/or local (non-profit) institutions in the development and/or promotion of mobile health interventions, (iv) managing expectations regarding the use of incentives, and (iv) identifying potential alternatives or complementary approaches to the use of chatbots, particularly for mental health.

## Electronic supplementary material

Below is the link to the electronic supplementary material.

Supplementary Material 1: Consolidated criteria for reporting qualitative studies (COREQ)

Supplementary Material 2: Topic guide

Supplementary Material 3: Thematic analysis checklist

## Data Availability

The datasets used and/or analysed during the current study are available from the corresponding author on reasonable request.

## Abbreviations

AI: Artificial intelligence
CORE-Q: Consolidated Criteria for Reporting Qualitative Research
CMD: Common mental disorder
HPB: Health Promotion Board
I-BMS: Integrative Body–Mind–Spirit
NCD: Non-communicable disease
SES: Socio-economic status
