# Supplementary material for: Exploring the potential of mobile health interventions to address behavioural risk factors for the prevention of non-communicable diseases in Asian populations: a qualitative study
Source: BMC Public Health. 2023 Apr 24;23:753. doi: 10.1186/s12889-023-15598-8 (PMC10123969; doi:10.1186/s12889-023-15598-8)
Supplement: Supplementary file 1 — Supplementary Material 1: Consolidated criteria for reporting qualitative studies (COREQ) [file 12889_2023_15598_MOESM1_ESM.doc]

**Consolidated criteria for reporting qualitative studies (COREQ)**

| **No. Item^1^** | **Guide questions/description** | **Reported on Page # and/or response** |
| --- | --- | --- |
| **Domain 1: Research team and reﬂexivity** |  |  |
| *Personal Characteristics* |  |  |
| 1. Interviewer/facilitator | Which author/s conducted the interview or focus group? | Page 6: “Focus groups were conducted online using a videoconferencing platform (Zoom Video Communications, Inc., San Jose, CA). Discussions were facilitated by two professional local moderators from the Facilitators Network Singapore, one male (lead) and one female (supporting), who were independent from the research team and had no personal interest in the research topic.” |
| 2. Credentials | What were the researcher’s credentials? E.g. PhD, MD | Interview facilitators: Professional local moderators from the Facilitators Network Singapore.  Research team directly involved in data analysis:  BF & JN (BSc –research assistants)  AS, JM & OC (PhD – postdoctoral researchers) |
| 3. Occupation | What was their occupation at the time of the study? | See item 2 |
| 4. Gender | Was the researcher male or female? | Interview facilitators: One male, one female.  Research team directly involved in data analysis: BF (female), JN (male), AS (female), JM (female) & OC (male) |
| 5. Experience and training | What experience or training did the researcher have? | Interview facilitators: extensive experience conducting and moderating focus group discussions.  Research team directly involved in data analysis: All members have completed several qualitative research courses as part of their bachelor and master’s studies. |
| *Relationship with participants* |  |  |
| 6. Relationship established | Was a relationship established prior to study commencement? | Page 6: “There were no prior relationships between the participants and the research team. However, some participants had taken part in previous focus group research moderated by the Facilitators Network Singapore.” |
| 7. Participant knowledge of the interviewer | What did the participants know about the researcher? e.g. personal goals, reasons for doing the research | Participants were aware that the purpose of the study was to explore perceptions, barriers, and facilitators to the use of digital health interventions for lifestyle behaviour change in Singapore. This was included in the informed consent.  Page 5: “Participants were eligible to join the focus groups if they were (…) (iv) able to provide informed consent |
| 8. Interviewer characteristics | What characteristics were reported about the interviewer/facilitator? e.g. Bias, assumptions, reasons and interests in the research topic | Participants were informed that the interviewers were professional facilitators and had no personal interest in the topic.  Page 6: “Discussions were facilitated by two professional local moderators from the Facilitators Network Singapore, one male (lead) and one female (supporting), who were independent from the research team and had no personal interest in the research topic.” |
| **Domain 2: study design** |  |  |
| *Theoretical framework* |  |  |
| 9. Methodological orientation and Theory | What methodological orientation was stated to underpin the study? e.g. grounded theory, discourse analysis, ethnography, phenomenology, content analysis | Page 7 |
| *Participant selection* |  |  |
| 10. Sampling | How were participants selected? e.g. purposive, convenience, consecutive, snowball | Page 5 |
| 11. Method of approach | How were participants approached? e.g. face-to-face, telephone, mail, email | Page 5 |
| 12. Sample size | How many participants were in the study? | Page 8 |
| 13. Non-participation | How many people refused to participate or dropped out? Reasons? | n/a (no dropouts) |
| *Setting* |  |  |
| 14. Setting of data collection | Where was the data collected? e.g. home, clinic, workplace | Page 6 |
| 15. Presence of non-participants | Was anyone else present besides the participants and researchers? | Page 6 |
| 16. Description of sample | What are the important characteristics of the sample? e.g. demographic data, date | Page 8 & table 1 |
| *Data collection* |  |  |
| 17. Interview guide | Were questions, prompts, guides provided by the authors? Was it pilot tested? | Page 6 |
| 18. Repeat interviews | Were repeat interviews carried out? If yes, how many? | n/a (no focus groups were repeated) |
| 19. Audio/visual recording | Did the research use audio or visual recording to collect the data? | Page 6 |
| 20. Field notes | Were ﬁeld notes made during and/or after the interview or focus group? | Page 6: “Two scribes (AA and RK) summarised participants’ comments using the chat function and took field notes during the discussions.” |
| 21. Duration | What was the duration of the interviews or focus group? | Page 6: “Focus groups were 90 minutes in duration, with 15 minutes allocated to introductions, guidelines, ground rules, and an ice breaker, 70 minutes allocated to the main discussion, and 5 minutes to close the session.” |
| 22. Data saturation | Was data saturation discussed? | Page 6 |
| 23. Transcripts returned | Were transcripts returned to participants for comment and/or correction? | n/a |
| **Domain 3: analysis and ﬁndings** |  |  |
| *Data analysis* |  |  |
| 24. Number of data coders | How many data coders coded the data? | Page 7 |
| 25. Description of the coding tree | Did authors provide a description of the coding tree? | Page 7 |
| 26. Derivation of themes | Were themes identiﬁed in advance or derived from the data? | Page 7 |
| 27. Software | What software, if applicable, was used to manage the data? | Page 7: “Transcripts were then uploaded to Atlas.ti V.9 to facilitate the analysis.” |
| 28. Participant checking | Did participants provide feedback on the ﬁndings? | n/a |
| *Reporting* |  |  |
| 29. Quotations presented | Were participant quotations presented to illustrate the themes/ﬁndings? Was each quotation identiﬁed? e.g. participant number | Pages 9-14 & Table 2 |
| 30. Data and ﬁndings consistent | Was there consistency between the data presented and the ﬁndings? | Pages 9-14 & Table 2 |
| 31. Clarity of major themes | Were major themes clearly presented in the ﬁndings? | Pages 9-14 |
| 32. Clarity of minor themes | Is there a description of diverse cases or discussion of minor themes? | Pages 9-14 |

^1^Developed from: Tong A, Sainsbury P, Craig J. Consolidated criteria for reporting qualitative research (COREQ): A 32-item checklist for interviews and focus groups. *Int J Qual Health Care*. 2007; 19: 349-57.
