# Supplementary material for: Exploring the potential of mobile health interventions to address behavioural risk factors for the prevention of non-communicable diseases in Asian populations: a qualitative study
Source: BMC Public Health. 2023 Apr 24;23:753. doi: 10.1186/s12889-023-15598-8 (PMC10123969; doi:10.1186/s12889-023-15598-8)
Supplement: Supplementary file 2 — Supplementary Material 2: Topic guide [file 12889_2023_15598_MOESM2_ESM.docx]

**A 15-Point Checklist Criteria for Good Thematic Analysis Process (Braun & Clarke, 2006)**

| **Process** | **No.** | **Criteria** | **Response** |
| --- | --- | --- | --- |
| Transcription | 1. | The data have been transcribed to an appropriate level of detail, and the transcripts have been checked against the tapes for ‘accuracy’. | Yes, several researchers were involved in the transcription process. |
| Coding | 2. | Each data item has been given equal attention in the coding process. | Yes, all quotations were reviewed to  generate coding. |
|  | 3. | Themes have not been generated from a few vivid examples (an anecdotal approach) but, instead, the coding process has been thorough, inclusive and comprehensive. | Yes, initial codes and themes were discussed thoroughly, with some researchers acting as ‘critical friends’ (Smith & McGannon, 2018). That is, reviewing candidate codes / themes and offering points for reflection and alternative explanations. |
|  | 4. | All relevant extracts for all each theme have been collated. | Yes, all extracts were collated in one  Atlas.ti file and each theme / sub-theme was checked for consistency. |
|  | 5. | Themes have been checked against each other and back to the original data set. | Yes, see item 3. |
|  | 6. | Themes are internally coherent, consistent, and distinctive. | Yes, see item 3. |
| Analysis | 7. | Data have been analysed rather than just paraphrased or described. | Yes, this was done through thorough  discussion among the researchers. It is worth noting that, based on our epistemology approach, qualitative methods aim to provide low-inference and straightforward descriptions of the phenomenon of interest. That is, within the surface or explicit meaning of the participants’ comments, rather than at the interpretative or latent level (more common in *reflexive* thematic analysis; Braun & Clarke, 2019). |
|  | 8. | Analysis and data match each other – the extracts illustrate the analytic claims. | Yes, to showcase this example quotes  were provided. |
|  | 9. | Analysis tells a convincing and well-organised story about the data and topic. | Yes, one of the purposes of using ‘critical friends’ was to ensure the analyses tell a convincing story of the data (one that answer the research questions) by revisiting the raw data under each theme and sub-theme. |
|  | 10. | A good balance between analytic narrative and illustrative extracts is provided. | Yes, see item 8. In addition to the quotes, a semantic description of themes and findings was presented in the results section. |
| Overall | 11. | Enough time has been allocated to complete all phases of the analysis adequately, without rushing a phase or giving it a once-over-lightly. | Yes. |
| Written report | 12. | The assumptions about thematic analysis are clearly explicated. | Yes, we state that our analyses were  grounded in an essentialist/realism  paradigm. |
|  | 13. | There is a good fit between what you claim you do, and what you show you have done – ie, described method and reported analysis are consistent. | Yes. |
|  | 14. | The language and concepts used in the report are consistent with the epistemological position of the analysis. | Yes. |
|  | 15. | The researcher is positioned as *active* in the research process; themes do not just ‘emerge’. | Yes, the researchers ‘made sense’ of  the data through thorough discussion. |

Braun, V., & Clarke, V. (2006). Using thematic analysis in psychology. Qualitative Research in Psychology, 3(2), 77–101. https://doi.org/10.1191/1478088706qp063oa
