# Supplementary material for: Exploring the potential of mobile health interventions to address behavioural risk factors for the prevention of non-communicable diseases in Asian populations: a qualitative study
Source: BMC Public Health. 2023 Apr 24;23:753. doi: 10.1186/s12889-023-15598-8 (PMC10123969; doi:10.1186/s12889-023-15598-8)
Supplement: Supplementary file 3 — Supplementary Material 3: Thematic analysis checklist [file 12889_2023_15598_MOESM3_ESM.docx]

**Part I: Focus Group Topic Guide**

Introductions and Ice breaker

1. Name
2. What do you do?
3. What do you most look forward to in your normal daily activities?
4. How has the coronavirus pandemic affected your everyday life?

Main Discussion

| Topic 1: Healthy Living | Relevant TDF Domains |
| --- | --- |
| Q1. What is your normal daily routine like? | Skills  Intentions |
| *Introduced in FGD3:*  Q2a. What is a healthy lifestyle to you? | Knowledge  Beliefs about consequences |
| Q2b. What stops you from making healthier lifestyle choices? | Beliefs about capabilities  Environmental context and resources  Social influences  Behavioural regulation |
| *Introduced FGD3 onwards:*  Q2c. If you had a choice; what could you do differently to improve what you are doing now? | Beliefs about capabilities  Intentions  Goals |
| Q3a. Name some health promotion activities/programs in Singapore | Knowledge |
| Q3b. What do you think of the health promotion activities/programs in Singapore? | Knowledge  Beliefs about consequences  Environmental context and resources |
| Q3c. How useful do you think they are? | Beliefs about consequences  Emotion  Behavioural regulation |
| Q4. What would be the most helpful source for supporting healthy lifestyle or mental health?  *Revised for FGD3 onwards to*:  If you were seeking support for healthy lifestyle including mental health where/who would you go to? | Knowledge  Social influences |
|  |  |
| Topic 2: Digital Interventions / Lifestyle and Chatbot Apps | Relevant TDF Domains |
| Q5 Name some lifestyle apps and technologies | Knowledge |
| Q5.1 What’s your experience of using health/lifestyle apps and technologies? | Environmental context and resources  Behavioural regulation |
| *Introduced FGD3 onwards:*  Q5b. What features would you like to see in these? | Beliefs about consequences  Behavioural regulation |
| Introduce conversational agent/chatbot coaching interventions and show example of a smartphone chatbot (*example below*) |  |
| Q6a. What are your first impressions of such apps with digital health coaches? | Beliefs about consequences  Intentions  Reinforcement |
| Q6b. How can we encourage people to use such an app long term? | Environmental context and resources  Social influences  Behavioural regulation |
| Q7. What are your views on sharing health data *(like heart rate, or step counts tracked by an activity tracker or a smartphone)?* | Knowledge  Intentions  Emotions |
| *Introduced FGD2 onwards:*  Q7b. Who would you be happy to share your personal data with? | Intentions  Emotions  Memory, Attention and Decision Processes |

Example of a smartphone chatbot

**
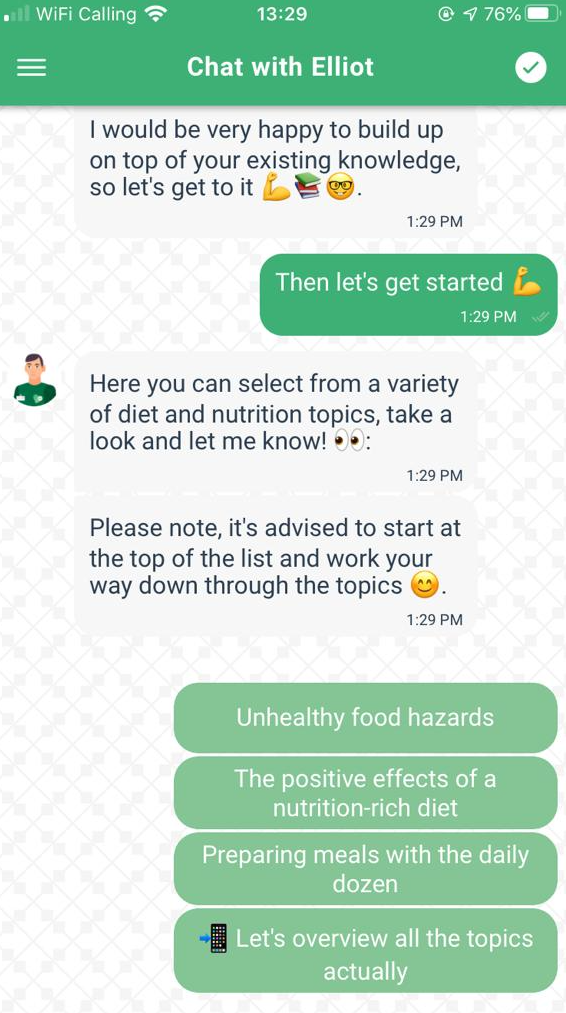

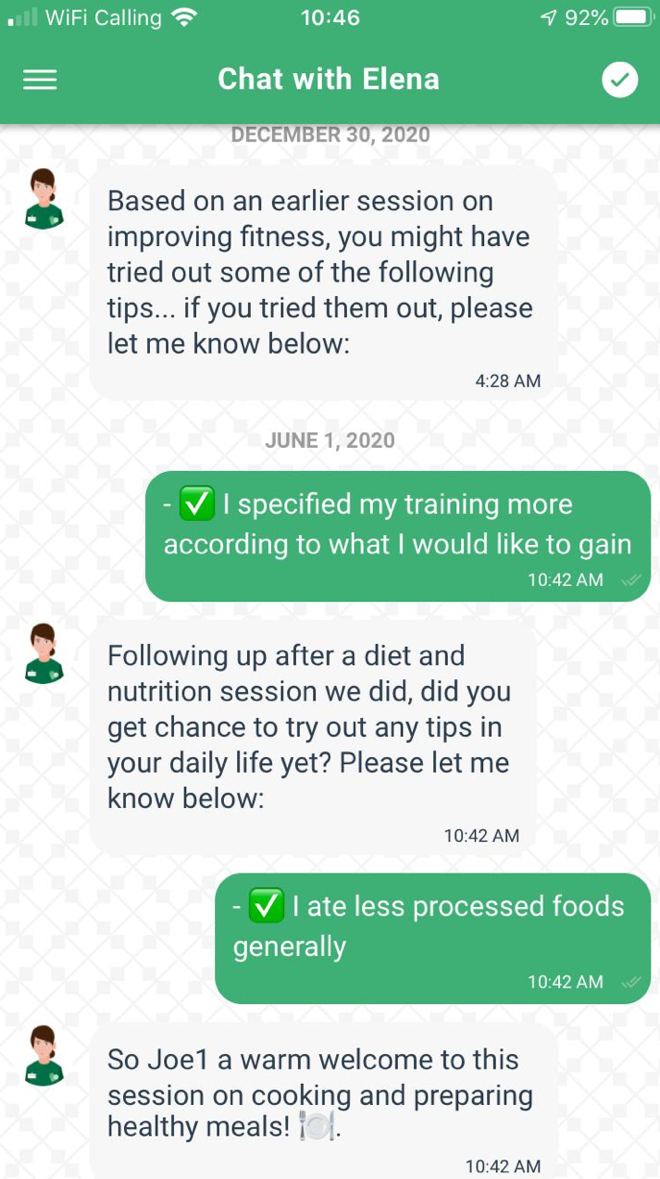
**

The Theoretical Domains Framework (v2) with definitions and component constructs (Cane et al., 2012).

| **TDF domains** | **Definition** | **Constructs** |
| --- | --- | --- |
| Knowledge | An awareness of the existence of something | Knowledge (including knowledge  of condition/scientific rationale)  Procedural knowledge  Knowledge of task environment |
| Memory attention and decision processes | The ability to retain information, focus selectively on aspects of the environment and choose between two or more alternatives | Memory  Attention  Attention control  Decision making  Cognitive overload/tiredness |
| Behavioural regulation | Anything aimed at managing or changing objectively observed or measured actions | Self-monitoring  Breaking habit  Action planning |
| Skills | An ability or proficiency  acquired through practice | Skills  Competence/ability/skill  assessment  Practice/skills development  Interpersonal skills  Coping strategies |
| Intentions | A conscious decision to perform a behaviour or a resolve to act in a certain way | Stability of intentions  Stages of change model  Trans theoretical model and  stages of change |
| Goals | Mental representations of outcomes or end states that an individual wants to achieve | Goals (distal/proximal)  Goal priority  Goal/target setting  Goals (autonomous/controlled)  Action planning  Implementation intention |
| Beliefs about consequences | Acceptance of the truth, reality, or validity about outcomes of a behaviour in a given situation | Beliefs  Outcome expectancies  Characteristics of outcome  expectancies  Anticipated regret  Consequents |
| Optimism | The confidence that things will happen for the best or that desired goals will be attained | Optimism  Pessimism  Unrealistic optimism  Identity |
| Beliefs about capabilities | Acceptance of the truth, reality or validity about an ability, talent or facility that a person can put to constructive use | Self-confidence  Perceived competence  Self-efficacy  Perceived behavioural control  Beliefs  Self-esteem  Empowerment  Professional confidence |
| Social / professional role and identity | A coherent set of behaviours and displayed personal qualities of an individual in a social or work setting | Professional identity  Professional role  Social identity  Identity  Professional boundaries  Professional confidence  Group identity  Leadership  Organisational commitment |
| Environmental context and resources | Any circumstance of a person’s situation or environment that discourages or encourages the development of skills and abilities, independence, social competence and adaptive behaviour | Environmental stressors  Resources/material resources  Organisational culture/climate  Salient events/critical incidents  Person × environment interaction  Barriers and facilitators |
| Social influences | Those interpersonal processes that can cause individuals to change their thoughts, feelings, or behaviours | Social pressure  Social norms  Group conformity  Social comparisons  Group norms  Social support  Power  Intergroup conflict  Alienation  Group identity  Modelling |
| Emotion | A complex reaction pattern, involving experiential, behavioural, and physiological elements, by which the individual attempts to deal with a personally significant matter or event | Fear  Anxiety  Affect  Stress  Depression  Positive/negative affect  Burn-out |
| Reinforcement | Increasing the probability of a response by arranging a dependent relationship, or contingency, between the response and a given stimulus | Rewards (proximal/distal, valued/not  valued, probable/improbable)  Incentives  Punishment  Consequents  Reinforcement  Contingencies  Sanctions |
